# Adenovirus-mediated delivery of bFGF small interfering RNA increases levels of connexin 43 in the glioma cell line, U251

**DOI:** 10.1186/1756-9966-29-3

**Published:** 2010-01-14

**Authors:** Biao Zhang, Xuequan Feng, Jinhuan Wang, Xinnu Xu, Hongsheng Liu, Na Lin

**Affiliations:** 1 Key Lab for Critical Care Medicine of the Ministry of Health, Affiliated Tianjin First Center Hospital, Tianjin Medical University, Tianjin, 300192, China; 2 Department of Neurosurgery, Affiliated Tianjin First Center Hospital, Tianjin Medical University, Tianjin, 300192, China

## Abstract

**Background:**

bFGF is an important growth factor for glioma cell proliferation and invasion, while connexin 43 is implicated in the suppression of glioma growth. Correspondingly, gliomas have been shown to have reduced, or compromised, connexin 43 expression.

**Methods:**

In this study, a bFGF-targeted siRNA was delivered to the glioma cell line, U251, using adenovirus (Ad-bFGF-siRNA) and the expression of connexin 43 and its phosphorylation state were evaluated. U251 cells were infected with Ad-bFGF-siRNA (100, 50, or 25 MOI), and infection with adenovirus expressing green fluorescent protein (Ad-GFP) at 100 MOI served as a control. Western blotting and immunofluorescence were used to detect the expression levels, phosphorylation, and localization of connexin 43 in U251 cells infected, and not infected, with Ad-bFGF-siRNA.

**Results:**

Significantly higher levels of connexin 43 were detected in U251 cells infected with Ad-bFGF-siRNA at 100 and 50 MOI than in cells infected with Ad-GFP, and the same amount of connexin 43 was detected in Ad-GFP-infected and uninfected U251 cells. Connexin 43 phosphorylation did not differ between Ad-bFGF-siRNA-infected and uninfected U251 cells. However, the ratio of phosphorylated to unphosphorylated connexin 43 in Ad-bFGF-siRNA cells was lower, and connexin 43 was predominantly localized to the cytoplasm. Using a scrape loading dye transfer assay, more Lucifer Yellow was transferred to neighboring cells in the Ad-bFGF-siRNA treated group than in the control group.

**Conclusion:**

To our knowledge, this is the first description of a role for connexin 43 in the inhibition of U251 growth using Ad-bFGF-siRNA.

## Background

Basic fibroblast growth factor (bFGF) is a heparin-binding growth factor that is secreted as a pleiotropic protein and can act on various cell types, including tumor cells. bFGF is hypothesized to have a critical role in the development of the nervous system [1], and for gliomas, the level of bFGF present has been shown to correlate with tumor grade and clinical outcome [2], bFGF has also been shown to be up-regulated in transformed glial cells and to be overexpressed in malignant gliomas [3]. bFGF exerts its cellular functions through the binding of four FGF receptors (FGFRs), all of which are receptor tyrosine kinases (RTKs). The binding of bFGF by FGFRs recruits and activates several signaling pathways [4]. Accordingly, down-regulation of bFGF using antibodies or antisense sequences has been shown to inhibit tumor cell tumorigenicity and metastasis [3,5,6]. A study by De Vuyst et al. also demonstrated a role for bFGF in the inhibition of gap junction (GJ) communication in the glioma cell line, C6, following exogenous expression of connexin 43 [7].

Connexin 43 (Cx43) is the predominant component of GJs which are composed of six connexin proteins and are differentially expressed in various cell types [8]. Several studies have demonstrated that Cx43 is one of the major GJ proteins expressed by astrocytes and glial cells [9], and in high-grade human gliomas, its expression is significantly reduced. Decreased expression of Cx43 observed in a variety of tumor types, including tumors of the central nervous system, can also affect GJ intercellular communication (GJIC) [10,11]. Restoration of GJIC by exogenous expression of Cx43 has reversed the transformed phenotype of certain tumor cells, including high-grade human gliomas [12,13]. In addition, susceptibility of the transfected glioma cells to apoptosis was enhanced in response to chemotherapeutic agents [14]. While it has been found that expression of Cx43 is inversely related to glioma cell proliferation and tumor grade [12,15,16], the specific regulatory mechanisms involving Cx43 in gliomas remains unclear.

In the present study, down-regulation of bFGF expression by a siRNA specifically targeted to bFGF is shown to significantly increase the expression of Cx43 without effecting the phosphorylation of Cx43 at S368 in the glioma cell line, U251.

## Methods

### Adenoviral vector construction

From four siRNA sequences that were designed for targeting bFGF, an optimal target sequence (5'-CGAACTGGGCAGTATAAACTT-3') was selected [17] and cloned into the plasmid vector, pGenesil-1. The siRNA expression cassette was subsequently excised from pGenesil-1 using *EcoR*I and HindIII and ligated into the linearized adenoviral shuttle vector, pGStrack-CMV. pGStrack-CMV-bFGF-siRNA was then co-transfected with the pAd vector backbone into DH5α bacteria for the recombinant generation of Ad-bFGF-siRNA, which was further amplified in HEK293 cells. Viral particles were purified using cesium chloride density gradient centrifugation.

### Cell culture and adenovirus infection

The human glioma cell line, U251, was maintained in Dulbcco's modified Eagle medium (DMEM) supplemented with 10% heat inactivated fetal bovine serum (FBS), 100 U/ml of penicillin, and 100 μg/ml of streptomycin in a humidified atmosphere containing 5% CO_2 _at 37°C. All media and serum were purchased from Gibcol.

U251 cells (1 × 10^5^) in serum-free DMEM were infected with Ad-bFGF-siRNA at 100, 50, and 25 MOI (MOI is calculate as PFU/cell numbers) in a humidified atmosphere containing 5% CO_2 _at 37°C. Infection with Ad-GFP at 100 MOI served as a control. Virus-containing medium was removed 8 h later and replaced with fresh DMEM medium containing 10% FBS. Cells were incubated for another 72 h, then mRNA or protein was extracted.

### MTT assay for cell proliferation

Cell proliferation was measured using MTT assay. 5 × 10^3 ^cells/well were seeded into 96 wells plate. After the adhesion of the cells, they were infected with Ad-bFGF-siRNA, meanwhile untreated cells and cells infected with Ad-GFP served as control and mock control. During consecutive seven days, 20 μl MTT solution (5 mg/ml) in PBS was added to each well for 4 h. After the culture medium was drained out, 150 μl of DMSO was added into each well. Absorbance of each well was measured on a microplate reader. Three duplicate wells were set up for each group.

### RT-PCR

Total RNA was extracted from cultured cells using TRizol reagents (Invitrogen, Carlsbad, CA, USA) according to the manufacturer's directions. First-strand cDNA was synthesized from total RNA(1 μg) using AMV reverse transcriptase (TaKaRa) with oligo(dT) primer at 42°C for 1 h in a 25 μl volume. RT product (2 μl) with cDNAs was mixed with bFGF or β-actin specific primers in a PCR buffer containing 2.5 mM dNTP, 2.5 mM MgCl_2 _and 1 U Taq polymerase (TaKaRa). PCR amplification was performed over 31 cycles (45 sec at 94°C, 60 sec at 60°C, and 45 sec at 72°C) to amplify bFGF, and over 25 cycles (30 sec at 94°C, 30 sec at 57°C, and 90 sec at 72°C) to amplify β-actin. Primers used for amplifying bFGF included: forward-5'-CACCATGGCAGCCGGCAGCATCA-3' and reverse-5'-TCAGCTCTTAGCAGACATTGG-3'. Primers used to amplify β-actin included: forward-5'-CCTCGCCTTTGCCGATC-3' and reverse-5'-GGATCTTCATGAGGTAGTCAGTC-3'. Amplified DNA fragments were separated in 2% agarose gels and visualized using ethidium bromide staining.

### Western blotting

Western blot analysis was performed on whole cell extracts obtained by direct dissolution of cells in culture flasks using a whole cell protein extract reagent according to the manufacturer's directions (PIERCE). Protein concentrations were determined using a bicinchoninic acid (BCA) protein assay kit with bovine serum albumin as a standard. Proteins (40 μg/lane) were separated on 12% SDS-PAGE gels and transferred onto polyvinylidene difluoride (PVDF) membranes. Membranes were blocked with 3% fat-free milk in PBST (0.2% Tween-20 in PBS, pH 7.6) then incubated with primary antibody for 18-24 h at 4°C. Membranes were subsequently incubated with secondary antibodies conjugated to horseradish peroxidase (1:5000) for 1 h at RT. Bound antibody was visualized using an Enhanced Chemiluminescence (ECL) western blot detection kit (Amersham Pharmacia Biotech). Primary antibodies used included: anti-bFGF (rabbit polyclonal, 1:1000, Santa Cruz), anti-Cx43 (rabbit polyclonal, 1:1000, Cell Signaling), anti-pCx43 for S368 (rabbit polyclonal, 1:1000, Cell Signaling), and anti-β-actin (mouse monoclonal, 1:1000, Santa Cruz).

### Immunofluorescence

U251 cells grown on cover slips were fixed with 4% paraformaldehyde for 15 min and permeabilized with 0.5% Triton X-100/PBS (Sigma-Aldrich) for 20 min. Cells were then washed twice with PBS and blocked in 3% bovine serum albumin (BSA) for 30 min prior to incubating the cells with primary antibodies recognizing Cx43 or p-Cx43 (S368) for 1 h in a humidified chamber. After several PBS washes, cells were incubated with tetraethyl rhodamine isothiocyanate(TRITC)-conjugated secondary antibodies for 1 h. After washing with PBS, cells were stained with Hoechst 33258 (Sigma-Aldrich) for 15 min and immunofluorescence was detected using a fluorescence microscope (Olympus).

### Scrape loading and dye transfer (SL/DT)

Levels of GJIC in control and treated U251 cells were determined using the scrape loading and dye transfer (SL/DT) technique with the fluorescent dye, Lucifer Yellow (LY), as a readout (Sigma). Briefly, U251 cells were seeded in 6-well plates and grown to confluency. After rinsing with PBS, cells were incubated with 0.05% (w/v) Lucifer Yellow in PBS. Scrape loading was performed using a surgical scalpel to draw several clear straight lines on the cell monolayer. After 5 min, the Lucifer Yellow solution was removed, cells were washed 4 times with PBS, and transfer of Lucifer Yellow was detected using an inverted fluorescence microscope.

### Statistical Analysis

All data were analyzed using SPSS 13.0 software. Significant differences were determined using either one-way analysis of variance (ANOVA) or a two-tailed Student t-test. A *p*-value < 0.05 was considered significant.

## Results

### Down-regulation of bFGF mRNA and protein in U251 cells using bFGF-targeted siRNA

**Figure 1:**
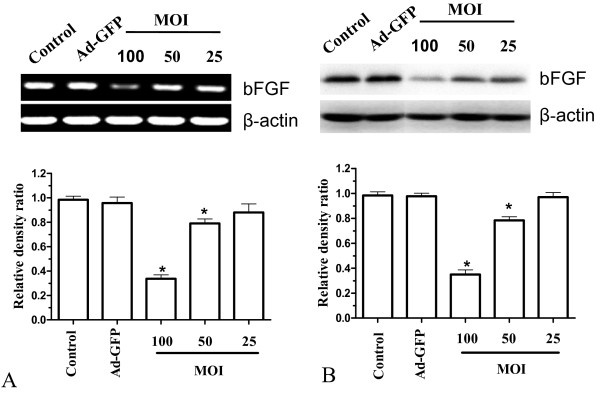
**Infection with Ad-bFGF-siRNA decreased the expression of bFGF mRNA and protein in U251 cells in a dose-dependent manner**. The level of bFGF mRNA (**A**) and protein (**B**) in control, Ad-GFP, and Ad-bFGF-siRNA-infected U251 cells as measured by RT-PCR and western blot. The upper panels include representative RT-PCR and western blot results, while the lower panels provide the relative band density ratios for bFGF mRNA and protein relative to β-actin (mean ± SD, n = 3) (*p < 0.05 vs. control).

**Figure 2:**
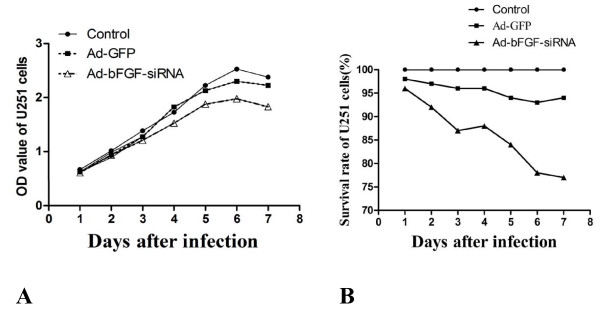
**Infection with Ad-bFGF-siRNA inhibited the proliferation of U251 cells**. Decrease of proliferation (**A**) and survival rate (**B**) in Ad-bFGF-siRNA treated U251 cells compared to untread U251 cells and Ad-GFP treated U251 cells. (mean ± SD, n = 3) (*p < 0.05 vs. control)

To examine changes in *bFGF *gene expression induced by adenoviral infection of bFGF-targeted siRNA, RT-PCR and western blot were performed. Both mRNA and protein levels of bFGF in Ad-bFGF-siRNA-infected U251 cells were dramatically reduced compared to bFGF levels in U251 infected with Ad-GFP or uninfected U251 (Fig. 1A, B). These results indicate that bFGF siRNA delivered by adenoviral infection can specifically suppress the expression of bFGF in U251 cells.Meanwhile, U251 cells, which were inhibited expression of bFGF using Ad-bFGF-siRNA, showed decrease of proliferation and survival rate compared to untread U251 cells and Ad-GFP treatment detected by MTT assay(Fig. 2A, B).

### The effect of bFGF-targeted siRNA on connexin 43 and phosphorylation of connexin 43 at PKC target site S368 in U251 cells

**Figure 3:**
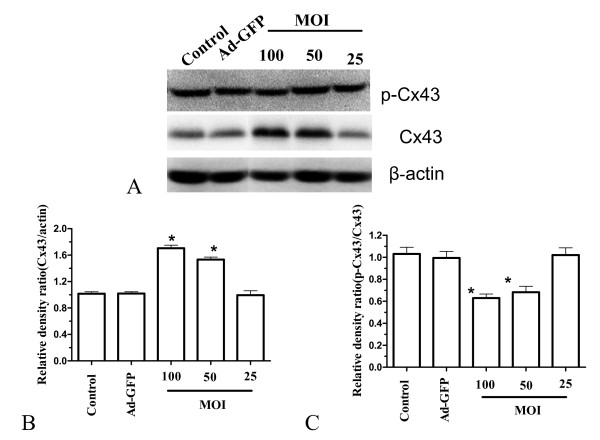
**Ad-bFGF-siRNA in U251 cells increases connexin 43 protein levels and no affect the level of p-connexin 43 at S368 site**. **A) **Expression of connexin 43 and p-connexin 43 at S368 site U251 cells infected with Ad-bFGF-siRNA and untreated U251 cells. A representative western blot is shown. **B) **Relative density values of Cx43 compared to β-actin from western blot analysis are provided. **C) **Relative density values of p-Cx43 compared to Cx43 from western blot analysis are provided. (mean ± SD, n = 3) (*p < 0.05 vs. control).

**Figure 4:**
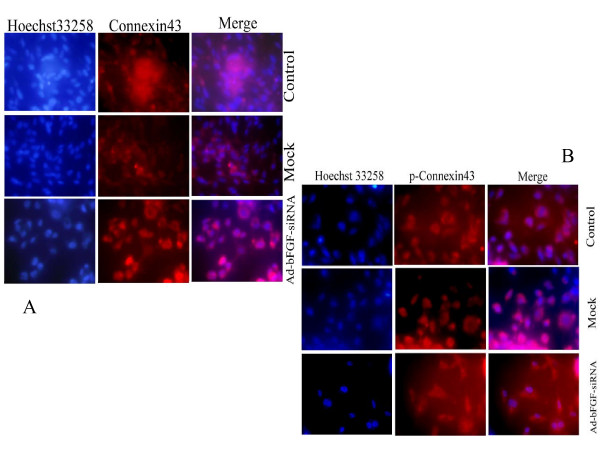
**Subcellular localization of Cx43 and p-Cx43 (S368) in Ad-bFGF-siRNA infected U251 cells**. **A) **Subcellular localization of Cx43 in U251 cells stained with anti-Cx43 antibody and with Hoechst 33258 staining to identify nuclei. **B) **Subcellular localization of p-Cx43(S368) in U251 cells stained with an anti-p-Cx43 antibody and Hoechst 33258 staining to identify nuclei.

Expression of Cx43 and phosphorylation of Cx43 at PKC target site S368 was detected in both bFGF-inhibited and uninhibited U251 cells using western blotting. In addition, the subcellular distribution of them in U251 cells was examined using indirect immunofluorescence. Western blotting revealed that inhibition of bFGF correlated with significantly higher levels of an immunoreactive 43 kDa band detected by a polyclonal Cx43 antibody relative to untreated U251 cells (Fig. 3A, B). While, down-regulation of bFGF did not affect phosphorylation of Cx43 at S368(Fig. 3A, C). Immunofluorescence studies identified Cx43 and p-Cx43 to be predominantly localized to the cytoplasm (Fig. 4A, B).

### Infection with Ad-bFGF-siRNA improves intercellular communication

**Figure 5:**
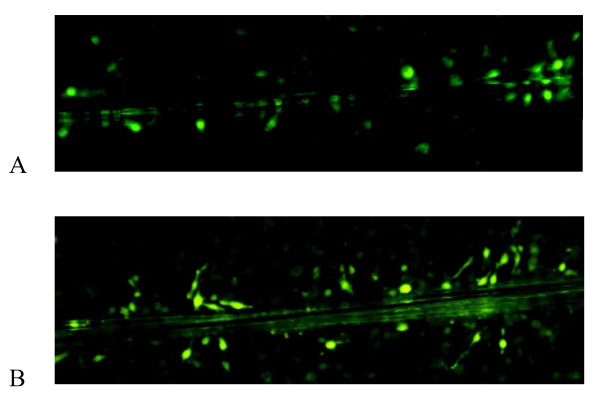
**Ad-bFGF-siRNA improves GJIC between U251 cells**. GJIC was assessed in U251 cells infected with Ad-bFGF-siRNA (100 MOI) for 48 h compared to untreated U251 cells using scrape loading dye transfer assays. **A) **In untreated cells, Lucifer Yellow was restricted to the cells at the border of the scraped line with only minimal transfer of Lucifer Yellow to neighboring cells. **B) **In Ad-bFGF-siRNA U251 cells, an increase in the transfer of Lucifer Yellow between cells was detected.

Scrape loading and dye transfer (SL/DT) assays were used to evaluate the permeability of GJs in U251 cells infected with Ad-bFGF-siRNA. Detection of the fluorescent dye, Lucifer Yellow (LY), showed a higher number of Ad-bFGF-siRNA-infected cells exhibited fluorescence than untreated U251 cells (Fig. 5). These results indicate that down-regulation of bFGF increased the GJIC between U251 cells.

## Discussion

The autocrine and paracrine signaling of bFGF makes it one of the most potent mitogenic factors for glial cell growth and differentiation. High levels of bFGF expression have also been associated with malignant grades of glioma, and in neoplastic astrocytes, bFGF stimulates the proliferation of astrocytoma cells. Conversely, inhibition of bFGF expression, or receptor binding of bFGF, has been demonstrated to inhibit glioma proliferation both *in vitro *and *in vivo *[18]. In the present study, infection with Ad-bFGF-siRNA down-regulated expression of bFGF in U251 cells, inhibited cell proliferation, and increased expression of Cx43. Huang have reported that Cx43 may suppress glioma proliferation by dowregulation of monocyte chemotactic protein 1(MCP-1)[19], the inhibitory effect of bFGF siRNA on U251 cell proliferation is at least partially due to the increased expression of Cx43, which may affect expression of other growth factors, such as down regulating MCP-1. However the correlation between downregulation of bFGF and inducion of Cx43 is still unclear, Ueki'study may provided some implicant, Ueki demonstrated in cortical astrocytes that epidermal growth factor (EGF) results in a decrease in the expression of Cx43 mRNA and protein and the decrease is associated with the receptor tyrosine kinase pathway, meanwhile the MEK inhibitor prevents EGF-stimulated down-regulation of Cx43 expression[20].

Immunofluorecence studies further demonstrated that increased expression of Cx43 localized primarily to the cytoplasm, with fewer molecules localizing to the perinucleus and sporadic plaques detected at the plasma membrane. In addition, dye transfer assays demonstrated that intercellular communication was improved for U251 cells infected with Ad-bFGF-siRNA. Consistent with data from other studies [21,22], it was observed that although localization of Cx43 was predominant at cytoplasm, the functions of GJIC mediated by Cx43 were normal.

Lack of Cx43 expression and aberrant localization of Cx43 have been associated with a lack of GJIC between tumor cells [23]. While gene mutations may play a role in deficient Cx43 expression, the precise mechanisms involved in decreased expression of Cx43 in tumor cells is still unclear. An increasing number of studies have shown that Cx43 can abnormally localize and accumulate in the cytoplasm in some cancer cell lines, including glioma cell lines. However, nuclear localization of connexin 43 has been reported in both *src *and *neu *oncogene-transformed rat liver epithelial cells [23]. Aberrant localization of Cx43 may also be associated with intact function of cytoskeletal elements [24].

Several studies have reported a role for Cx43 in both physiological and pathological conditions, although with contrasting results [25-27]. There are two mechanisms that have been postulated to explain the observed discrepancies. For example, Cx43 may directly mediate intercellular communication to permit the transport of factors that inhibit or enhance cell growth, or alternatively, Cx43 may affect GJs directly [28,29]. Based on studies in a rat glioma cell line, regulation of glioma growth is proposed to be more dependent on the behavior of connexins than the activity of GJIC [30]. Therefore, it is possible that Cx43 may effect tumor growth independently of GJ formation. Despite these insights, further studies are necessary to define the precise role of Cx43 in glioma cell communication and growth.

## Competing interests

The authors declare that they have no competing interests.

## Authors' contributions

BZ participated in study design, performed experiments and and drafted the manuscript. XF carried out experiments. JW participated in study design and revised manuscript. XX participated in study design and helped to draft the manuscript. HLcarried out statistical analyses NL performed experiments and helped to draft the manuscript. All authors approved the final manuscript.
